# Insights into Salinity Tolerance in Wheat

**DOI:** 10.3390/genes15050573

**Published:** 2024-04-29

**Authors:** Zechao Zhang, Zelin Xia, Chunjiang Zhou, Geng Wang, Xiao Meng, Pengcheng Yin

**Affiliations:** Ministry of Education Key Laboratory of Molecular and Cellular Biology, Hebei Research Center of the Basic Discipline of Cell Biology, Hebei Collaboration Innovation Center for Cell Signaling and Environmental Adaptation, Hebei Key Laboratory of Molecular and Cellular Biology, College of Life Sciences, Hebei Normal University, Shijiazhuang 050024, China; zhangzechao1117@163.com (Z.Z.); xiazelino@126.com (Z.X.); cjzhou@hebtu.edu.cn (C.Z.); gengwang@hebtu.edu.cn (G.W.); mengxiao2012@163.com (X.M.)

**Keywords:** wheat, salt stress, breeding, sodium

## Abstract

Salt stress has a detrimental impact on food crop production, with its severity escalating due to both natural and man-made factors. As one of the most important food crops, wheat is susceptible to salt stress, resulting in abnormal plant growth and reduced yields; therefore, damage from salt stress should be of great concern. Additionally, the utilization of land in coastal areas warrants increased attention, given diminishing supplies of fresh water and arable land, and the escalating demand for wheat. A comprehensive understanding of the physiological and molecular changes in wheat under salt stress can offer insights into mitigating the adverse effects of salt stress on wheat. In this review, we summarized the genes and molecular mechanisms involved in ion transport, signal transduction, and enzyme and hormone regulation, in response to salt stress based on the physiological processes in wheat. Then, we surveyed the latest progress in improving the salt tolerance of wheat through breeding, exogenous applications, and microbial pathways. Breeding efficiency can be improved through a combination of gene editing and multiple omics techniques, which is the fundamental strategy for dealing with salt stress. Possible challenges and prospects in this process were also discussed.

## 1. Introduction

Soil salinity causes significant abiotic stress on agricultural crop productivity globally, jeopardizing the yield of crops [1]. The primary factors contributing to soil salinity include climatic variations, geological and hydrological conditions, and human activities, particularly excessive irrigation. As a result, managing salinization is challenging, and the extent of salinized areas of land may continue to increase [2]. With an estimated 840 million people at risk of hunger by 2030, it is imperative to improve crop cultivation strategies to feed growing populations [3]. Common wheat (*Triticum aestivum* L., AABBDD, 2n = 42) is a widely cultivated crop globally, serving as a staple food for many nations [4]. However, the limited area of arable land and adverse climate conditions pose challenges to meeting this demand without a new agricultural revolution. Studies show that, for every unit increase in salinity, seeds, spike, 1000-grain weight and economic yield are significantly reduced in both salt-sensitive and salt-tolerant wheat varieties [5]. There are a large number of marginal saline lands around the world, such as coastal areas, that have the potential for production if engineered, salt-tolerant wheat is feasible [6]. An understanding of the impact of high salinity on wheat’s metabolic, physiological, biochemical, morphological properties, and gene expression is crucial for achieving this objective.

Soil salinization leads to the excessive accumulation of salt ions, primarily Na^+^ and K^+^, in plant cells, resulting in severe ion toxicity. Furthermore, salt stress can induce secondary stresses, such as osmotic stress and oxidative damage, which can disrupt metabolic processes. Plant responses to osmotic stress and ion toxicity, such as ion balance regulation, osmotic balance regulation, and reactive oxygen clearance are well understood in *Arabidopsis thaliana*, rice (*Oryza sativa*) and other species [7,8].

The salt tolerance levels of different plant species vary significantly [9]. Among cereal crops, wheat exhibits a moderate level of salt tolerance, being less tolerant than barley (*Hordeum vulgare*) but more tolerant than rice [10]. An understanding of the salinity tolerance molecular mechanism in wheat is limited due to its large and redundant genome, although research on salt tolerance regulation has progressed in other crops [11,12]. In addition, the demand for salt-resistant wheat varieties in agricultural is rising, making it of great theoretical and practical significance to clone new salt-tolerant genes, analyze their genetic and molecular mechanisms, and create germplasm resources for the breeding of high-yield and salt-tolerant cultivars of wheat. This review highlights recent research on the molecular regulation mechanism of wheat salinity tolerance and discusses the potential of new technologies in enhancing wheat salinity tolerance in order to provide a theoretical basis for improving wheat breeding to increase salinity tolerance.

## 2. Physiological and Molecular Cognition of Salt Stress Responses

### 2.1. Overview of the Effects of Salt on Wheat and Physiological Basis

The primary stresses caused by salt on plants are mainly ionic toxicity and osmotic stress. Osmotic stress directly affects crop growth by reducing the expansion of root tips and young leaf cells. On the other hand, the effects of ion stress on crop growth appears relatively late. In plants with Na^+^ transport capacity, the impact of ion stress is usually not as significant as that of osmotic stress [13].

The impact of salt stress on above-ground growth can be divided into two stages, as follows: an initial rapid response to increased external osmotic pressure and a subsequent slow response to the accumulation of Na^+^ in leaves [14]. During the first stage, new shoot growth is hindered, while in the second stage, older leaves exhibit accelerated aging. Notably, in cereals during the initial stage, a significant reduction in tillering and spikelet numbers is observed under salt stress. The decrease in cellular water potential caused by osmotic stress can limit water utilization in plants. When exposed to salt stress, both salt-tolerant and salt-sensitive varieties experience a decline in relative water content (RWC), albeit to a lesser extent in salt-tolerant wheat varieties [15].

Salt stress also induces the production of reactive oxygen species (ROS) such as superoxide anion (O^2−^), hydrogen peroxide (H_2_O_2_) and hydroxyl radical (·OH). Excessive accumulation of ROS causes oxidative stress, which further affects the growth and development of crops by destroying membrane lipids and causing cell toxicity through protein oxidation [16]. NADPH oxidase (NOXs), also known as respiratory burst oxidase (Rboh), is a major producer of ROS in plants. NOX/Rboh phosphorylation plays a key role in the Ca^2+^-ROS signaling pathway, and Ca^2+^ accumulation in the cytoplasm is a prerequisite for the activation of NOXs/RBOHs [17].

#### 2.1.1. Enzyme Reaction under Salt Stress

To mitigate the adverse effects of salt stress, plants employ various responses, including sensory mechanisms, mineral nutrient absorption, ion balance regulation, osmoregulation, the clearance of ROS, etc. Salt-tolerant plants develop antioxidant mechanisms by activating antioxidant enzymes such as ascorbate peroxidase (APX), catalase (CAT), superoxide dismutase (SOD), and peroxidase (POD) [18]. SOD converts O^2−^ into low-toxic H_2_O_2_ and non-toxic O_2_ to maintain ROS homeostasis, preventing the generation of more active OH^−^. CAT has a strong affinity for H_2_O_2_, which can catalyze the decomposition of H_2_O_2_ into H_2_O and O_2_. In wheat, ten CATs have been identified to combat various environmental stresses [19]. POD acts as a protective enzyme that inhibits H_2_O_2_ production in the cell wall, while glutathione (GSH) peroxidase (GPX) mainly converts oxidized glutathione to reduced glutathione. APX utilizes ascorbic acid as a substrate to convert H_2_O_2_ into water, thus alleviating the damage of free radicals in plant cells. Together, these enzymes form an antioxidant system that synergistically regulates the redox balance and eliminates ROS in plants [20].

#### 2.1.2. Response of Organelles under Salt Stress

**Figure 1:**
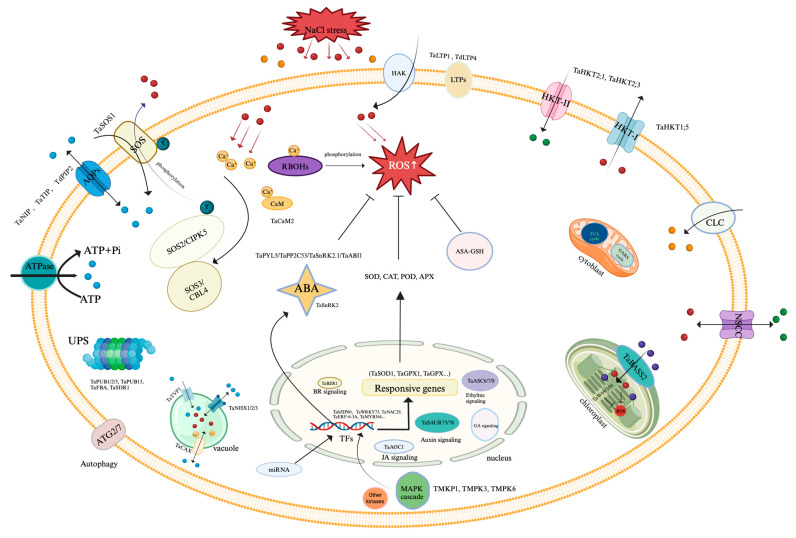
Known salt stress responses and signaling pathways in wheat: (1) the SOS signaling pathway is the most classical signaling pathway under salt stress, which in wheat consists of TaSOS3/TaCBL4, TaSOS2/TaCIPK5 and TaSOS1 [23], and transports Na^+^ out of the cell by sensing cellular Ca^2+^ signals. Ca^2+^ is the second messenger of the cell, and the Ca^2+^-CBL-CIPK network maintains homeostatic balance in plants, including the antioxidant system and the ABA signaling pathway; (2) the HKT gene family are Na^+^-selective transporters, TaHKT1;5-D, Nax1 and Nax2 maintain a high K^+^/Na^+^ ratio in wheat by limiting Na^+^ transport into the cell [14], and other channels, including chloride channel proteins, water-selective channel proteins and NSCCs, are also involved in the response of wheat to salt stress; (3) salt stress induces cellular ABA accumulation and the wheat ABA receptor TaPYL5 can regulate ROS homeostasis under abiotic stress through the TaPP2C53/TaSnRK2.1/TaABI1 signaling pathway [24]. The activation of ABA synthesis and signaling pathways enhances the expression of salt-tolerant genes and maintains cellular ion homeostasis. Other hormones such as JA, BR and GA also play important roles; (4) in transcriptional regulation, TaFDL2-1A-TaNCED2/TaSOD1/TaGPX1, TaCDPK-TabZIP60-TaNCED1/TaLEA1 and other pathways respond to salt stress [25] and NAC, MYB, WRKY, AP2/ERF, etc. improve salt tolerance of wheat by regulating the expression of stress-related genes; (5) at the post-transcriptional translation stage, overexpression of *TaPUB1/2/3*, *TaPUB15*, *TaFBA-2A* and *TaSDR1* improves salt tolerance in plants, suggesting that wheat may be involved in protein degradation through ubiquitin-mediated pathways [26]; (6) *TaATG2/7* was involved in the response of wheat to salt stress, suggesting that salt stress-induced deleterious substances may be degraded by autophagy; and (7) TMKP1 interacts with TMPK3 and TMPK6 in vivo, and *TMKP1* overexpression in Arabidopsis under salt stress has a higher germination rate [27], suggesting that wheat MAKP may play an active role in regulating the response of plant cells to salt and osmotic stress.

Chloroplasts play a crucial role in photosynthesis in green plants. When plants experience stress, their photosynthetic efficiency can be affected, and the ROS production in leaf greens increases under severe conditions. Maintaining proper cell homeostasis is essential for the normal function of chloroplasts. Pyruvate serves as a precursor for the synthesis of abscisic acid (ABA) and other metabolites, with BILE ACID/SODIUM SYMPORTER 2 (BASS2) being responsible for the transport of pyruvate into chloroplasts [18]. In Arabidopsis, *bass2-1* obstructs plastidal isopentenyl diphosphate (IPP) synthesis. The overexpression of *BASS2* in Arabidopsis can restore the defective pyruvate transporter phenotype of *bass2-1*. Furthermore, TaBASS2 enhances the salt tolerance of Arabidopsis by suppressing the expression of *ABA INSENSITIVE 4* (*ABI4*), indicating that pyruvate transporters in chloroplasts are involved in plant salt stress [21]. Through RNA-seq, two GPX genes, *W106* and *W69*, localized in chloroplasts, were identified. These genes encoded proteins capable of reducing H_2_O_2_ and t-butyl hydroperoxide (t-BHP) in vitro (Figure 1). Arabidopsis overexpressing these genes exhibited increased tolerance to NaCl, H_2_O_2_ and ABA. Following salt treatment, the transcription levels of genes like *SOS1*, *RbohD*, and *ABI1*/*ABI2* from wheat were altered in transgenic Arabidopsis lines compared to the wild type, suggesting that chloroplast-localized TaGPXs may regulate ROS homeostasis and ABA signaling [22].

The endoplasmic reticulum (ER) plays a key role in metabolite synthesis and transport, protein synthesis, modification, and protein-quality control [28]. When plants face environmental changes such as heat, drought, salt, and pests and diseases, they adapt to these changes by regulating protein biosynthesis. The ER in wheat seedling leaves can alleviate cell stress by degrading misfolded proteins and reducing ribosome translation under salt stress [28].

The mitochondrial metabolic process plays a role in enhancing salt tolerance, likely through the energy it provides for ion transport [29]. When plants are exposed to salt stress, certain key enzymes involved in the tricarboxylic acid (TCA) cycle within wheat mitochondria are partially inhibited [30]. However, plants have various metabolic pathways related to the TCA cycle, such as the malic acid to pyruvate pathway and the γ-aminobutyric acid (GABA) diversion pathway (Figure 1). Research involving the measurement of proteins and metabolites associated with the TCA cycle, GABA shunt, and mitochondrial membrane transport has shown that under salt stress conditions, there is a decrease in pyruvate uptake in mitochondria along with an increase in GABA shunt activity [31]. The increased GABA activity can provide another carbon supply for the TCA cycle through succinic acid production rather than the mtPC and mtPDC steps [31]. Various mitochondrial membrane proteins in durum wheat, including mitochondrial phospholipase A2 (PLA2), plant uncoupling protein (PUCP), endometrial anion channel (PIMAC) and mitochondrial potassium channel (PmitoKATP), participates in the response to salt stress. However, further investigation is needed to determine the functions of these proteins in hexaploid wheat [32].

### 2.2. Ion Transportation under Salt Stress

Toxic ion absorption by plant roots is primarily controlled by transporters located in the plasma membrane. Aquaporins (AQPs), also known as water-selective channel proteins, mediate rapid transmembrane water flow during plant growth (Figure 1). The overexpression of wheat *TaNIP* (an *AQP* gene) in transgenic Arabidopsis led to higher salt tolerance [33]. Similarly, overexpression of the wheat aquaporin gene *TaAQP8* was shown to enhance the transgenic tobacco’s salt tolerance [34]. Additionally, the overexpression of *TdPIP2;1*, a wheat *AQP* gene, increases the tolerance of transgenic wheat to both drought and salt stress [35]. Under salt and drought stress, *TaTIP4;1* has emerged as a key positive regulator of seedling growth and seed germination by affecting the water relationship, ROS balance, proline, and Na^+^ accumulation through stimulating the stress response genes [36].

Lipid transfer proteins (LTP) bind acyl chains and facilitate the phospholipids’ transfer between membranes (Figure 1). NaCl treatment increased the expression of *TaLTP1* gene, which mitigated cell wall damage from abiotic stress, potentially aiding in wheat’s adaptation to water stress. The transcription of *TaLTP1* gene is regulated by MYC and MYB during salt and dehydration stress in wheat [37]. The overexpression of *TdLTP4* in Arabidopsis can promote plant growth under NaCl, ABA, methyl jasmonate (MeJA), and H_2_0_2_ stress conditions [38]. Furthermore, the overexpression of *TdLTP2* in Arabidopsis improved plant tolerance to salt, ABA and salicylic acid (SA) by mobilizing the antioxidant system [39].

Accumulation of Na^+^ in plant tissues can inhibit K^+^ absorption from soil. Maintaining an appropriate Na^+^/K^+^ ratio in plant is crucial for plant salt tolerance. Bread wheat can maintain a low Na^+^/K^+^ ratio in the whole plant except the root; the *Kna1* locus on chromosome 4DL is associated with this trait [40]. In durum wheat, recombinant obtained by recombining the 4D (from bread wheat) and 4B chromosomes confirmed that the exclusion of Na^+^ in the shoot and the decrease in Na^+^/K^+^ ratio was controlled by a single locus, *Kna1*. This results in lower Na^+^/K^+^ ratio in flag leaves, leading to increased grain yield and biomass under salt stress [41]. High-affinity K^+^ Transporter (HKTs) plays a key role in Na^+^ efflux. *Nax1* and *Nax2* were identified that were associated with the Na^+^ exclusion trait using durum wheat and *Triticum monococcum* introgression parent hybrids. *Nax1* on chromosome 2AL, which is not homologous to the *Kna1*, codes for *HKT1;4-A2*, regulating Na^+^ unloading from the xylem of the root and leaf sheath; *Nax2* on chromosome 5AL, homologous to *Kan1*, codes for *HKT1;5-A* and *HKT1;5-D*. *Nax1* and *Nax2* can reduce the Na^+^ concentration in wheat leaves by 50% and 30%, respectively, with a combined reduction of 60% [42]. Studies have shown that *HKT1;5-D*, a key Na^+^ transporter in wheat, plays a crucial role in Na^+^ tolerance in natural hexaploid wheat by removing excess Na^+^ from xylem ducts, thereby keeping it below toxic levels in photosynthetic tissues [10]. Additionally, HKT2 is involved in regulating the absorption of Na^+^ in K^+^-deficient plants to compensate for the K^+^ deficiency, and the downregulation of *TaHKT2* (*TaHKT2;1* and *TaHKT2;3*) in the salt-tolerant wheat genotypes confer a tolerance to salt stress in wheat [43]. Salt stress can lead to cytosine methylation in genotype-specific and tissue-specific ways, resulting in the downregulating of *TaHKT2;1* and *TaHKT2;3* expression in shoots and roots [44]. However, the *TaHKT1;4* gene does not appear to be influenced by DNA methylation [44]. During salt stress, non-selective cation channels (NSCC) regulated by ROS, which is produced in chloroplasts, mainly mediates K^+^ efflux in bread wheat mesophyll [45]. K^+^ absorption and transport are mainly facilitated by K transporters and K channel proteins, including three families of transporters (KUP/HAK/KT, HKT and CPA families) and two families of ion channel proteins (Shaker and KCO/TPK families). For example, *TaHAK13* promotes K^+^ absorption, particularly in a low-potassium (K) medium in Arabidopsis [46] (Figure 1).

When cells accumulate excessive Na^+^, the transport of Na^+^ into the vacuole is an effective response for plants (Figure 1). This process not only reduces the cytosolic toxicity, but also acts as an osmoregulator to facilitate water absorption under salt stress. The Na^+^/H^+^ reverse transporter (NHX) family are involved in both Na^+^ efflux and the compartmentation of Na^+^ into vacuoles. NHX activity is regulated by an electrochemical H^+^ concentration gradient across the vacuolar membrane, which is generated by V-H^+^-ATPase or V-H^+^-PPase (H^+^-translocating inorganic pyrophosphatase). Transgenic wheat plants that overexpress the vacuolar Na^+^/H^+^ reverse transporter gene exhibited decreased Na^+^ levels in leaves, enhanced salt tolerance, and increased yield in saline–alkali soil [47]. The overexpression of the *NHX1* gene and the vacuolar H^+^-PPase coding gene (*VP1*) can improve salt tolerance in plants. Arabidopsis plants with overexpression of the vacuolar membrane H^+^-PPase gene (*TVP1*) or *TaNHX1* showed greater resistance to high NaCl concentrations and water scarcity compared to wild types and accumulated more Na^+^ and K^+^ in leaf tissues [48]. Transgenic tomato plants with *TaNHX2* also exhibited improved salt stress compared to wild-type plants [49]. The transformation of *SeVP1* and *SeVP2* from *S. europaea* into wheat and Arabidopsis resulted in improved performance under high-salt and low-nitrogen (N) conditions compared to wild-type plants [50]. Wheat *TaVB* (*vacuolar H^+^-ATPases subunit B*) gene can improve plant salt tolerance. Arabidopsis overexpressed with *TaVB* has higher V-H^+^-ATPase activity, root length, germination rate and overall salt tolerance [51]. The vacuolar type H^+^-ATPase (V-type H^+^-ATPase) gene, *W36*, from wheat, can enhance the salt tolerance of overexpressing Arabidopsis plants through osmotic reaction [52]. The V-H^+^-ATPase subunit in wheat plays a key role in improving plant salt tolerance [53].

The salt overly sensitive (SOS) pathway plays important roles in Na^+^ efflux. In this pathway, Ca^2+^ is sensed by SOS3/CBL4 and interacts with serine/threonine protein kinase SOS2/CIPK24 to activate SOS2. Once phosphorylated, the SOS2-SOS3 complex activates the plasma membrane Na^+^/H^+^ transporter SOS1/NHX7, facilitating the transportations of Na^+^ out of cells [54]. Essentially, the active efflux of Na^+^ from wheat root epidermal cells is mediated by SOS1-like homologs that are activated by H+-ATPase in the plasma membrane. Mutations in components of the SOS pathway often result in a significant decrease in salt tolerance in plants. When subjected to salt stress, wheat lines with a high expression level of the *TaSOS1* gene exhibit improved salt tolerance through the development of a higher Fv/Fm value, more robust root system and water potential [55]. TaSOS1 regulates plasma membrane Na^+^/H^+^ transport, with TaSOS1-A1 playing a more crucial role in Na^+^ exclusion than either TaSOS1-D1 or TaSOS1-B1 in bread wheat [23]. Through the treatment of three wheat species with salt stress, it is found that the SOS pathway of ion homeostasis is conserved across different species [56] (Figure 1). Chloride ions are essential micronutrients in higher plants, contributing to various functions including the PSII electron transport chain in photosynthesis, osmoregulation, and enzymes activation [57]. However, some experiments have found that high Cl^−^ may exhibit toxicity comparable to high Na^+^ levels [58]. Plant chloride channel (CLC) has been shown to regulate nutrient transport, stress tolerance and exhibit conserved voltage-gated chloride channel functions [59] (Figure 1). In Arabidopsis, *AtCLC-a* gene is located on the vacuole membrane and acts as a NO_3_^−^/H^+^ exchanger. *AtCLC-c* is involved in stomatal motility and salt tolerance. *AtCLC-d* mediates the transport of anions such as Cl^−^ or NO_3_^−^ [60]. While twenty-three *TaCLC* genes have been identified in wheat, their functions remain unclear; however, the expression of wheat *TaCLC* family members can be induced under conditions of low nitrogen and salt stress [59].

### 2.3. Signal Transduction under Salt Stress

#### 2.3.1. Ca^2+^ Signal

As a second messenger in cells, calcium is involved in signal transduction for various abiotic stresses and growth regulation [61]. Plants possess several types of Ca^2+^-sensing proteins, including calcineurin B-like (CBL) protein, calcium-dependent protein kinases (CDPKs) and calmodulin-like proteins (CML) (Figure 1). CBL protein interacts with a set of serine/threonine kinases known as CBL-interacting protein kinases (CIPKs) or SnRK3s. CBL protein plays an important role in calcium sensing and contributes to the decoding of Ca^2+^ by forming a complex signaling network with the CIPKs. The CBL-CIPK regulatory network has been shown to be implicated in various physiological processes, such as salt stress in Arabidopsis and rice; however, there is limited knowledge of this network’s role with respect to wheat [62]. Studies have shown that CIPK9 and CBL3 collaborate to maintain K^+^ homeostasis under low-K^+^ stress in Arabidopsis. Additionally, CBL10 directly interacts with the K^+^ channel protein (AKT1) and regulates its activity in Arabidopsis K^+^ homeostasis regulation under ion stress independently of CIPK [63]. A wheat *TaCIPK14* gene that encodes a CIPK, increases the seed germination rate, chlorophyll and sugar contents, and catalase activity in transgenic tobacco under salt stress, while decreasing Na^+^ content, H_2_O_2_, and malondialdehyde (MDA) content [64]. ROS induced by salt stress triggers downstream signaling pathways by influencing Ca^2+^ concentrations. The overexpression of *TaCIPK29* from the wheat CIPK gene family enhanced the salt tolerance in tobacco by participating in the regulation of cation and ROS homeostasis [65]. *TaCIPK25* negatively regulates the salt response in wheat, with its expression being regulated by *TaWRKY9*. Under high-salinity conditions, the expression of *TaCIPK25* in roots was significantly downregulated. The overexpression of *TaCIPK25* leads to the hypersensitivity reaction and excessive accumulation of Na^+^ in transgenic wheat [66]. *TdCBL6*, a CBL gene from wild emmer wheat (*Triticum dicoccoides*), when expressed in Arabidopsis, reduces H_2_O_2_ content and ion leakage (EL), thereby improving the salt tolerance of transgenic lines by mitigating membrane damage and improving photosynthetic efficiency. The *TdCBL6* gene also participates in the response to low K^+^ stress by regulating the low K^+^ signaling pathway [67].

Several members of the wheat *CDPK* gene family are associated with stress resistance. *CPK7* and *CPK12* are present in the common ancestors of many grass species. *TaCPK7* and *TaCPK12* exhibit distinct responses to low temperatures, drought, and salt stress due to differences in promoter subfunctionalization. These proteins aid in stress resistance in wheat by participating in H_2_O_2_ and ABA responses [68]. Silencing of the *TaCDPK27* gene in wheat leads to the accumulation of excess ROS and increased salt-induced programmed cell death (PCD), demonstrating that *TaCDPK27* is necessary for salt-tolerance regulation in wheat seedlings [69]. TaCDPK5/TaCDPK9-1 can be auto-phosphorylated and phosphorylate the TabZIP60 protein in a Ca^2+^ dependent manner. The phosphorylation of TabZIP60, catalyzed by TaPP2CA116/TaPP2CA121 and TaCDPK5/TACDPK9-1 during salt stress, may play a key role in wheat [70]. TabZIP60 is implicated in the regulation of salt tolerance through ABA synthesis, facilitated by its interaction with TaCDPK30 in wheat [25].

CaM and CML are the most effective sensing proteins involved in Ca^2+^ signaling, although their roles remain unclear in wheat. Researchers identified 15 *TaCaMs* and 113 *TaCMLs* from the wheat genome and found that *TaCaMs* may play an important role in coping with abiotic organisms, including salt stress, and that the overexpression of *TaCAM2-D* in Arabidopsis increased its salt tolerance [71].

Glycogen synthase kinase (GSK) is involved in various physiological process and signal transduction pathways in plants. The binding property of wheat TaGSK1 protein to CaM is dependent on Ca^2+^, and the overexpression of *TaGSK1* in Arabidopsis enhances salt tolerance by reducing osmotic pressure and Na^+^ content [72]. *TaSK5*, a novel GSK3/SGG kinase from winter wheat, responds to a variety of abiotic stress factors. The ectopic expression of *TaSK5* in Arabidopsis confers salt tolerance to the transgenic plants by stimulating the expression of a set of osmotic/drought stress response genes [73] (Figure 1).

Calreticulin (CRT), initially identified in animals, has been demonstrated to play a role in Ca^2+^ storage and in the regulation of cellular Ca^2+^ homeostasis in plants [74]. Three *CRT* genes from wheat were cloned and introduced into tobacco, and the *TaCRT1* overexpressed tobacco exhibited notably enhanced tolerance to salt stress [75].

#### 2.3.2. Transcription Factors

At the molecular level, transcription factors (TFs) regulate gene expression. During abiotic stress, they regulate relevant genes’ expression positively or negatively within the intricate signal regulatory network of cells, ultimately ensuring the maintenance of plant growth and development. TFs like WRKY, NAC, bZIP, VQ, MYB and AP2/ERF are known to be directly or indirectly associated with stress response. Some transcription factors related to salt tolerance in wheat have been identified and studied (Table 1).

WRKY TFs participate in plant stress responses. Examples include *TaWRKY24*, *TaWRKY2*, *TaWRKY13*, *TaWRKY19*, *TaWRKY44* and *TaWRKY46*, which have been shown to improve the salt tolerance to wheat [76]. Additionally, tobacco plants that overexpress *TaWRKY10* have been found to exhibit enhanced tolerance to salt and drought [77]. WRKY TFs can directly or indirectly impart flavonoid synthesis by activating related enzyme genes or other regulators, thereby enhancing plant salt tolerance [93].

The AP2/ERF TFs play a critical role in modulating gene expression during abiotic stress, primarily through the MAPK and ethylene signaling pathways [94]. The AP2/ERF family is categorized into five groups: RAV, DREB, AP2, Soloist, and ERF. A study examining 543 *AP2/ERF* genes in wheat identified 43 genes involved in salt stress response genes [95]. The overexpression of *TaERF-6-3A* or *TaDREB3-A1* in Arabidopsis increases sensitivity to salt stress [78]. *TaERF3* positively regulates wheat’s adaptive responses to salt and drought stress by activating stress-related genes, making it a promising target for improving abiotic stress tolerance in cereals including wheat [79].

MYB TFs possess a highly conserved myb DNA-binding domain, typically featuring one to four replicates [80]. These TFs are divided into four subfamilies based on the number of replicates present. The R2R3-MYB is the largest subfamily in the plant, encompassing 223 genes, including 8 genes responsive to salt stress in wheat [82]. Various MYB TFs such as *TaMBY344*, *TaODORANT1*, *TaMYB32*, *TaMYB33*, *TaMYB64*, *TaMYB73*, *TaMYB86B*, *TaMYBsdu1* and *TaMYB19* enhance salt tolerance in transgenic tobacco or Arabidopsis plants [80,81,82,83].

The plant NAC family (NAM, ATAF1, ATAF2 and CUC2) is a major TF family in plant stress responses [96]. Specifically, *TaNAC29* responds to salt stresses by engaging the antioxidant enzyme systems and ABA signal pathway. *TaNAC2*, *TaNAC2a* and *TaNAC67* improve the transgenic Arabidopsis’s tolerance to salt stress [84].

The bZIP TFs can regulate tolerance of salt stress by binding to the ABRE element, which is responsive to ABA [97]. The overexpression of *TabZIP60* or *TaNCED2* in wheat results in increased salt tolerance through the ABA pathway [85]. *TabZIP15* and *TabZIP14-B* are involved in the regulation of salt tolerance in wheat [86], and *TabZIP8*, *9* and *13* are involved in the ABA biosynthesis under salt stress through the regulation of *TaCDPK9*-1 [87]. *TaFDL2-1A* promotes the expression of *TaNCED2*, *TaGPX1* and *TaSOD1* to enhance ABA biosynthesis, salt tolerance, and ROS clearance in wheat [88].

Researchers have identified 225 bHLH-coding genes in the wheat genome. There are few studies on the bHLH family in wheat associated with salt stress. *TabHLH1* regulates plant salt tolerance through ABA-related pathways [89]. The overexpression of *TabHLH39* significantly enhanced the tolerance of Arabidopsis to salt stress at the seedling stage [90].

Zinc finger proteins (ZnFPs) are common transcription factors found in eukaryotes. Among them, C_2_H_2_ type zinc finger proteins have been proven to be involved in plant development and stress resistance [98]. Overexpression of *TaZnFP* in Arabidopsis can enhance drought tolerance and salt tolerance of the transgenic plants, which indicates that *TaZnFP* could be a key regulator in plant responses to abiotic stress [91]. Wheat *TaZNF* significantly improved the salt tolerance of transgenic Arabidopsis by enhancing the excretion of Na^+^ [92].

#### 2.3.3. Posttranslational Control of Proteins

1. Plant kinases and phosphorylation

Sucrose nonfermenting1 related protein kinases (SnRKs) are pivotal in abiotic stress signaling in plants, mediating the phosphorylation of target proteins to regulate downstream signaling pathways [99]. The SnRK family in plants is categorized into three subfamilies: SnRK3, SnRK2, and SnRK1, among which SnRK2 is crucial for plant responses to abiotic stresses, notably osmotic and salt stress [100]. The overexpression of *TaSnRK2.8*, *TaSnRK2.4* and *TaSnRK2.3* in Arabidopsis significantly enhances tolerance to salt, freezing and drought stress by reducing the water-loss rate, increasing the relative water content and photosynthetic potential, and enhancing cell membrane stability [101,102]. Similarly, the overexpression of *TaSnRK2.9* improves tobacco’s tolerance to salt and drought stress, as well as its root length, seed germination and survival rate by enhancing ROS clearance, the specific SnRK–ABF interaction and ABA-dependent signal transduction [103].

Plant mitogen-activated protein kinases (MAPKs or MPKs) are critical mediators of a broad spectrum of biological processes [104]. Specific isoforms, including *TaMPK6*, *TaMPK16*, *TaMPK17*, *TaMPK14*, *TaMPK12;1* and *TaMPK4* are implicated in mediating plant responses and tolerance to multiple stress factors via distinct MAPK cascade modules [105]. Wheat *TMPK3*, functionally analogous to *AtMPK3*, plays a pivotal role in enhancing plant salt tolerance [106]. In eukaryotes, MAP kinase phosphatases (MKPs) act as negative regulators of MAPKs [27]. Under conditions of salt and osmotic stress, *TMKP1* is upregulated in salt-sensitive wheat varieties. TMKP1 interacts with TMPK6 and TMPK3 in vivo, suggesting a role in modulating plant cellular responses to osmotic and salt stress [27]. The overexpression of *TMKP1* in Arabidopsis leads to an increased germination rate under salt stress while its expression is ectopic in an *mkp1* [107] (Figure 1).

Serine/threonine protein kinases (STKs) participate in signal transduction pathways, including those relating to abiotic stress in plants [108]. A wheat STK gene (*TaSTK*) was cloned through the comparison of gene expression between salt-tolerant and salt-sensitive mutant lines in wheat, with its function being preliminarily characterized [109]. The overexpression of *TaSTK* in Arabidopsis led to enhanced salt stress tolerance, evidenced by increased root growth in salt-containing media [109].

Phosphatidylinositol regulates the recruitment and activity of signaling proteins within cell membranes in plants [110]. Phosphatidylinositol (PI) 4-kinases (PI4Ks) catalyze the production of PI 4-phosphate (PI4P), a key precursor of regulatory phosphoinositide in Arabidopsis. The overexpression of a stress-inducible type II *PI4K* gene from wheat, *TaPI4KIIγ*, is associated with enhanced salt and drought tolerance, primarily through an improvement in root growth [111].

Reversible phosphorylation is a mechanism regulating the signal transduction for both normal developmental and environmental stress [112]. A significant proportion of dephosphorylation events within cells are mediated by protein type 1 phosphatase (PP1) in Arabidopsis [113,114].

2. Ubiquitination

Protein modification by ubiquitin (Ub) and ubiquitin-like proteins (Ubls) constitutes a pivotal post-translational regulation mechanism in eukaryotes, critically influencing numerous biological pathways [115]. In plant cells, 26S and 20S proteasomes mediate ubiquitin-dependent and non-ubiquitin-dependent proteolysis, respectively. Particularly, the 20S proteasome in wheat roots is posited to play a significant role as a protease under conditions of salt stress, accumulating in the roots [116].

The ubiquitin-26S proteasome system (UPS) orchestrates selective protein degradation through a tripartite process involving Ub-ligating (E3), Ub-conjugating (E2), and Ub-activating (E1) enzymes, ensuring the precise regulation of protein levels [115].

The ubiquitin-associated domain (UBA) plays a significant role in plant responses to abiotic stress. *TaUBA* expression is induced by drought and salt stress, acting as a negative regulator in Arabidopsis [117]. E3 ubiquitin ligases, essential for tagging proteins for degradation, are categorized into two main types based on conserved domain presence: single-subunit types (U-box, RING, and HECT) and multi-subunit types (CUL4-DDB, CUL3-BTB, APC, and SCF) [118]. The Plant U-box (PUB) E3 proteins regulate protein degradation in response to plant stress through a proteolytic mechanism involving multiprotein E3 ubiquitin ligases. The overexpression of *TaPUB15* in rice exhibited improved salt tolerance, maintaining a low Na^+^/K^+^ ratio under salinity stress, and significantly upregulated salt-stress-related genes [119]. The overexpression of *TaPUB1* in wheat enhanced antioxidant enzyme activity, contributing to salt tolerance [120]. Overexpression *TaPUB2* and *TaPUB3* in Arabidopsis bolstered ABA stress response and salt tolerance [121]. The F-box protein, a core subunit of the Skp1-cullin-F-box (SCF) complex E3 ligase, is crucial in plant growth, biotic and abiotic stress responses, and hormone signaling pathways. In tobacco overexpressing *TaFBA1*, an F-box gen, increased salt tolerance through enhanced antioxidant activity and Na^+^/K^+^ ion homeostasis [122]. *TaFBA-2A* negatively affected jasmonic acid (JA) biosynthesis in wheat and may mediate TaOPR2 degradation via the ubiquitin-26S proteasome pathway [26]. SDIR1 is a RING E3 ubiquitin ligase with a C3H2C3 type domain. The overexpression of *TaSDIR1* shows higher salt tolerance at seedling and germination stage in wheat, while *TaSDIR1* silenced wheat shows higher salt sensitivity, indicating that TaSDIR1 positively enhances salt stress tolerance in wheat [123] (Figure 1).

#### 2.3.4. Autophagy

Autophagy is executed through the formation of autophagosomes, which are pivotal for sequestering dysfunctional or misfolded proteins and transporting them to the vacuole for degradation [124]. Researchers have observed that salt treatment disrupts homeostasis in root cells, leading to the accumulation of ROS, which in turn triggers autophagy and programmed cell death (PCD) [125]. Exposure to NaCl stress results in the accumulation and augmentation of autophagosomes in the roots and leaves of wheat seedlings [125]. It has been shown that the expression of *ATG2* and *ATG7* is induced by salt. The silencing of *ATG2* or *ATG7* disrupts ionic balance, precipitating excessive ROS production and heightening PCD levels [126] (Figure 1).

#### 2.3.5. Hormones

Plant hormones are crucial regulators in plants. Abscisic acid (ABA), JA, auxin, gibberellin (GA), brassinosteroid (BR), cytokinin (CTK), and ethylene are among the hormones that play significant roles in modulating responses to salt stress (Table 2).

Salt tolerance in wheat, enhanced through salt acclimation, can be further improved by ABA priming, which facilitates better water retention, protects the photosynthetic electron transport chain, and boosts carbon assimilation in salt-acclimated plants [139]. Wheat plants rapidly accumulate ABA in response to abiotic stress, enhancing tolerance by modulating the plant antioxidant system [16]. ABA enhances salt tolerance in wheat seedlings by reducing transpiration flow, maintaining Na^+^ homeostasis, and increasing antioxidant enzyme activity [140]. ABA’s primary mechanism involves regulating the activity of PP2C phosphatases [141]. The ABA receptor (RCAR /PYL/PYR) binds to PP2C phosphatases forming a complex that jointly inhibits PP2C activity in Arabidopsis [141]. This inhibition leads to phosphorylation of SnRK2 protein kinase through the phosphorylation of downstream transcription factors, such as ABRE-binding proteins, AREB, or ABF in response to ABA, activating or inhibiting their functions. Additionally, transcription factors including WRKY, NAC, AP2, and bZIP are involved in ABA signaling pathways [85]. The overexpression of *TaABL1*, a novel ABI-like TF gene, confers increased drought, salt and cold stress tolerance in Arabidopsis and tobacco [127]. The ABA pathway can facilitate ROS clearance in wheat. TaPYL5, a wheat ABA receptor, regulates ROS equilibrium under abiotic stress via TaPP2C53/TaSnRK2.1/TaABI1 signaling pathways [24] (Figure 1). In wheat, 33 *TaASRs* (abscisic acid-, stress- and ripening-induced genes) have been identified [142], most of which respond primarily to salt and low temperature stress [143]. *TaASR1-D* overexpression promotes grain yield and salt tolerance in wheat under salt stress by enhancing antioxidant capacity [16]. The plant steroid hormone gene *TaBZR1* activates *TaNCED3* (the ABA biosynthesis gene), *TaGPX2*, and *TaGPX3* (ROS clearance genes), promoting ABA synthesis and improving wheat salt tolerance [128].

JA is pivotal in abiotic stress regulation in plants. MeJA significantly alleviates salinity’s negative effects by enhancing plant transpiration rates, RWC, biomass, stomatal conductance, photosynthetic rate, chlorophyll contents, oxidative stress indicators, internal CO_2_ concentrations, the K^+^/Na^+^ ratio, and enzymatic activity (APX, CAT, SOD and POD) [144]. Under salt stress, the expression of *LOX*, *AOC*, *ACX*, and other crucial genes in the JA synthesis pathway, increases in plants. Exogenous JA elevates POD activity in wheat seedlings, thereby improving salt tolerance [129]. *TaAOC1*, through the α-linolenic acid metabolic pathway and *TaWRKY75-A* by promoting JA synthesis via the JA biosynthesis pathway, enhances salt tolerance in wheat and Arabidopsis, respectively [130].

Under salt stress, auxin maintains ion homeostasis by enhancing the germination rate, shoot weight, and the development of adaptive roots [145]. Auxin biosynthesis can be induced under abiotic stress, leading to a reduction in lateral root formation, directing roots away from stress areas [114]. In response to high salt stress, Arabidopsis root architecture undergoes significant remodeling through alter the accumulation and redistribution of auxin. *AtWRKY46* contributes to salt stress adaptation by modulating lateral root development via ABA signaling and auxin pathways [146]. *SAUR*s (small auxin upregulated RNAs) are influenced by both auxin and environmental factors. The overexpression of *TaSAUR75* enhances drought and salt tolerance by increasing root length and survival rates in Arabidopsis [131]. *TaSAUR78* regulates the interaction with *TaVDAC1* (voltage-dependent anion channel) to enhance abiotic stress tolerance in Arabidopsis [132]. Moreover, the overexpression of *TaLAX3-1B* (*like AUX3*) alters the pore size in tobacco, thereby improving its salt tolerance [133].

GA plays a pivotal role in regulating dormancy and seed germination in plants [147]. The exogenous application of GA has been shown to enhance soybean growth recovery and alleviate the detrimental effects of salt stress [148]. Treatment with GA during seed initiation improves resilience to high salinity, manifesting in enhanced photosynthetic pigments, an expanded leaf area, accelerated plant growth, and increased grain weight and quality [149]. Studies indicate that GA pre-treatment modulates growth and yield in genetically diverse wheat varieties under salt stress by altering ion distribution and gas exchange properties [150]. The observed increase in grain yield and viability in saline conditions can be attributed to hormonal homeostasis and the GA-driven regulation of ion uptake and distribution in buds and roots [148].

BRs, a class of phytohormones, are critical in stress resistance [151,152,153]. The foliar application of BRs as exogenous plant growth regulators has improved wheat growth under various conditions [154]. The gene *TaD11-2A*, involved in BR biosynthesis, influences salt tolerance at the seedling stage in rice and wheat, and is linked to grain size and yield enhancement [134]. Under salt stress, the expression of BRASSINAZOLE-RESSTANT1 gene (*TaBZR1*), a key transcription factor in wheat BR signaling, is significantly increased. The overexpression of *Tabzr1-1D* (function acquisition *TaBZR1*-mutated protein) enhances salt tolerance in wheat by activating genes related to ABA synthesis and ROS scavenging [128]. Additionally, the synergy between BR and arbuscular mycorrhizal (AM) fungi may improve salt tolerance in wheat by maintaining cellular ion homeostasis and regulating Na^+^/H^+^ antiporters [135]. Genome-wide studies of BR-related genes in wheat, maize, barley, and sorghum have explored their roles in salt tolerance, suggesting that *TaGRAS19-4B*, *TaBRD1-2A.1* and *TaMADS22/47/55-4B* may contribute to wheat’s resistance to salt [136].

Cytokinin treatment at seed initiation under salt stress elevates germination rates through enhanced water-use efficiency and photosynthesis, thereby promoting growth and yields in wheat [155,156].

Ethylene, synthesized as ethephon, enhances Glu utilization under salt stress by affecting photosynthetic potential, thereby attenuating the Glu-mediated inhibition of photosynthesis to mitigate the impact of salt stress on wheat growth [157]. Ethylene signaling plays essential roles in mediating plant abiotic stress responses and development. The overexpression of wheat *TaACO1* (coding aminocyclopropane-1-carboxylate oxidase), a key enzyme in ethylene biosynthesis, results in the increased expression of AtMYB15 in transgenic Arabidopsis, while repressing *AtCBF3*, *AtCBF1* and *AtRAB18*, consequently rendering the plants more susceptible to salinity [137]. Integrated transcriptomic and proteomic analyses have identified ethylene-dependent responses to salt stress involving chaperone synthesis, ROS scavenging, and carbohydrate metabolism pathways [158]. Amino acid penetrases (AAPs), a category of membrane proteins, including the *TaAAP* gene, respond to salt stress. The overexpression of *TaAAP1* in transgenic wheat enhances salt tolerance by upregulating the ethylene synthesis gene (*TaACS6*/*TaACS7*/*TaACS8*), leading to increased ethylene accumulation [138]. Furthermore, salt stress has been shown to significantly elevate the expression of ethylene-related genes in wheat, suggesting that ethylene response factors (ERFs) may play a pivotal role in mediating ethylene signaling and enhancing salt tolerance in wheat [159].

## 3. Strategies to Enhance Salt Tolerance of Wheat

### 3.1. Breeding

#### 3.1.1. Conventional Crossbreeding

**Figure 2:**
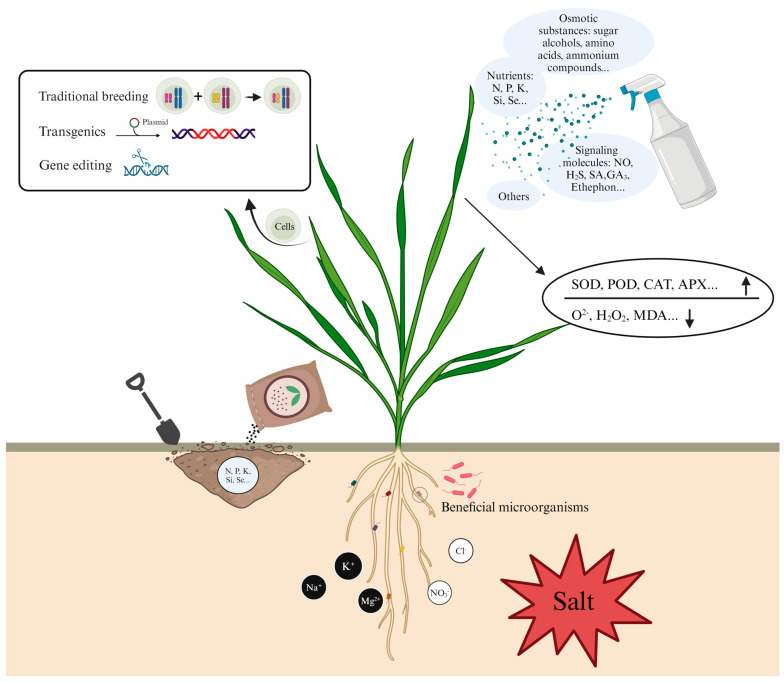
Strategies to improve salt tolerance in wheat: (1) acquisition of parental salt tolerance traits through crossbreeding, transfer of salt tolerance genes into wheat bodies through transgenesis and alteration of salt-sensitive genetic information through gene editing; (2) alteration of enzyme activity and hormone levels in wheat by exogenous application such as osmotic substances, nutrients and signaling substances; (3) changing soil physico-chemical properties through fertilizer application; and (4) the enrichment of beneficial microorganisms in the root system was conducive to the improvement of wheat salt tolerance.

Crossbreeding, a conventional method in plant breeding, has significantly contributed to the development of desirable traits in wheat, exemplified by the dwarfing characteristics introduced during the green revolution, which not only enhanced lodging resistance but also markedly boosted yield [160] (Figure 2). The Triticeae tribe, which encompasses around 350 species of wheat’s wild relatives, exhibits extensive genetic diversity, especially in salt tolerance, which varies widely among its members [161]. Genera such as *Elytrigia* and *Thinopyrum* originate from coastal areas, while *Leymus* species thrive in highly saline and alkaline soils. A halophytic relative of wheat, tall wheatgrass (*Thinopyrum ponticum*), stands out as one of the most salt-tolerant monocotyledons, and is capable of surviving in conditions as saline as seawater [162]. This group includes numerous halophytes; for instance, sea barleygrass (*Hordeum marinum*) and tall wheatgrass (e.g., *Thinopyrum* spp.) exhibit greater salt tolerance than common wheat [163]. The variance in salt tolerance among these species presents an opportunity to enhance the salt tolerance of wheat through crossbreeding, highlighting the potential of wild species to improve wheat’s resilience to salinity. Unlike disease resistance, the attribute of salt tolerance in wheat is governed by multiple genes, making its assessment in breeding more challenging [164]. Since the 1980s, intergeneric hybridization has been employed to transfer salt resistant genes to common wheat from Triticeae species [161]. However, 25 years post the initial experiments, the resulting new varieties have not yet been widely adopted by farmers due to challenges such as yield and environmental adaptability [164].

Interspecific hybridization between varieties possessing desirable traits and wheat often encounters the recombination barrier due to incompatibility. Shanrong No. 3 (SR3), a wheat variety that exhibits both salt tolerance and high yield, was developed through the use of asymmetric somatic cell fusion technology. This variety, with Jinan 117 serving as the maternal line and *Thinopyrum longiculosa* as the paternal line, underwent successful field trials in saline–alkali soil in 1998. The trials demonstrated SR3’s superior performance in both yield and salt resistance, showing a 30–40% increase in production compared to the control in saline–alkali soil conditions [165].

Currently, crossbreeding remains a method under investigation and utilization by numerous researchers for wheat breeding [166,167]. With ongoing advancements in science and technology, the integration of assistive technologies offers various optimization strategies for crossbreeding, thereby expanding the potential and applicability of crossbreeding techniques [168].

#### 3.1.2. New Breeding Techniques

While conventional crossbreeding has yielded substantial achievements, several challenges persist, including a relatively low utilization rate of wheat-related plant chromosomal translocation lines in breeding programs. Additionally, the integration of desirable traits, such as salt tolerance, is often hindered by issues like distant cross-incompatibility and hybrid sterility. To overcome these obstacles, wheat breeding should be integrated with modern molecular biology techniques such as gene editing, transgenic technologies, and molecular marker-assisted breeding. These approaches can enhance breeding efficiency and enable the precise manipulation of target traits (Figure 2).

Molecular marker-assisted breeding combines traditional genetic breeding with modern molecular biology to cultivate superior crop germplasms [169]. This approach, known as marker-assisted selection (MAS), is employed globally to enhance a range of traits in wheat, particularly for the improvement of key economic characteristics [170]. A new wheat germplasm was developed by conducting four backcrosses using MAS for the *Nax1* and *Nax2* genes with various Turkish genotypes and Australian salt-tolerant varieties as the recurrent parents. This method produced progeny combinations carrying genes for salt tolerance [171]. Given that plant salt tolerance often encompasses multiple traits and mechanisms, MAS enables the early selection of target traits, facilitates multiple selections within a relatively short timeframe, and allows for the stacking of tolerance components from diverse genetic resources. This strategy could be a potent means to substantially enhance plant salt tolerance. Compared to conventional field evaluation and phenotypic selection, the advantages of MAS offer a promising pathway to develop crops with improved salt tolerance [172]. Although numerous QTLs for crop salt tolerance have been identified across various species, the development of commercial cultivars or breeding lines with enhanced salt tolerance through MAS has been limited [173].

Identifying reliable loci for salt tolerance and its associated traits has been challenging due to the complex nature of soil salinity and the difficulty in differentiating the effects on infiltration and the salt-specific impact on leaf area production [174]. However, QTL mapping has emerged as a crucial tool in unraveling the genetics underlying complex plant traits, especially with the gradual enhancement of wheat genome data [175]. QTLs with additive (a) and a x a (aa) epistatic effects were identified and their salt treatment interactions (at and aat) were characterized at the seedling stage. A correlation between chlorophyll content and all biomass traits, as well as some physiological traits, emphasize that the pathways for Na^+^ and K^+^ accumulation are genetically distinct [176]. Numerous molecular markers linked to genes or QTLs influencing significant traits have been identified, offering potential indirect selection criteria to enhance breeding efficiency through MAS [173].

Transgenic technology serves as a crucial optimization strategy in wheat breeding, enhancing disease resistance, resilience, and productivity through the introduction of exogenous genes. This technology plays a significant role in advancing the breeding of wheat crops, aiming to increase yield and quality in response to evolving environmental conditions and human demands.

### 3.2. Exogenous Application

The breeding process is lengthy and its outcome uncertain. In contrast, the exogenous application of certain plant growth regulatory compounds represents the most direct approach to enhancing the salt tolerance of wheat [177] Such substances encompass plant nutrients, antioxidants, signaling molecules, and osmoprotectants, among others [178] (Figure 2). Interactions with these external compounds induce the plant to enter a specific physiological state, wherein various defense mechanisms are activated [179]. This activation enables the crops to respond more swiftly and effectively to salt stress.

At the physiological level, osmoregulation is a crucial adaptive mechanism that enhances drought or salt tolerance, enabling plants to maintain turgor pressure under stress conditions. Extensive evidence from various in vitro and in vivo studies, employing physiological, biochemical, genetic, and molecular methodologies, underscores the significant roles of osmotic substances. These substances include sugars and sugar alcohols (such as trehalose, D-ononitol, sorbitol, fructans, and mannitol), amino acids (like proline and exobases), and ammonium compounds (including O-choline sulfate, β-alanine betaine, polyamines, glycine betaine, and dimethyl sulfopropionate) in bolstering plant resistance to both drought and salt stress [180].

Adequate plant nutrition is fundamental for crop yields. Beyond supporting plant growth and functionality, nutrients also positively influence the mitigation of salt stress effects on wheat crops [181]. Excessive salinity leads to increased absorption of Cl^−^ and Na^+^, while the uptake of Zn^2+^, Ca^2+^ and K^+^ decreases, resulting in nutrient deficiencies that impair normal plant growth and metabolism [182]. Key nutrients for plants include K, N, phosphorus (P), magnesium (Mg), silicon (Si), selenium (Se) and zinc (Zn), among others [183]. Potassium is known to enhance the salt tolerance of wheat by activating respiration enzymes, boosting photosynthetic efficiency, and aiding in osmotic regulation [184]. Si improves wheat growth under salt stress by modulating the flow and partitioning of Na^+^, Cl^−^ and mineral ions, as exogenous application, establishing Si as a valid strategy with which to enhance the salt tolerance of crops at the field level [185]. Furthermore, the application of Se can mitigate wheat damage by reducing ROS during salt pressure [186].

Non-enzymatic antioxidants are crucial in reducing the content of ROS in plants, including ascorbic acid (AsA), GSH, tocopherol, and other compounds. Salt stress significantly exacerbates lipid peroxidation, prompting a notable increase in the activities of antioxidant enzymes in plants [167]. The application of AsA to wheat roots can mitigate the adverse impacts of salt stress on certain wheat cultivars by enhancing endogenous AsA levels and CAT activity, maintaining ion homeostasis and improving photosynthetic capacity [187]. Melatonin is recognized as a potential antioxidant in plants. Under specific salt concentrations, the application of melatonin (200 µM) can enhance wheat yield by reducing H_2_O_2_ accumulation and regulating the polyamines of wheat seedlings [188].

While the phytohormones and antioxidants within plants are known to have specific signaling roles, this discussion will concentrate on the role of externally applied signaling molecules such as NO, H_2_S, H_2_O_2_, and CO. Exogenous NO has been found to decrease Na^+^ concentrations while increasing K^+^ concentrations in seeds. This effect is achieved by enhancing soluble sugar content, respiration rate, ATP synthesis, and reducing MDA and H_2_O_2_ levels, thus maintaining the K^+^/Na^+^ balance during seed germination under salt stress and ultimately improving germination rates [189]. Furthermore, NO can mitigate salt damage in wheat seedlings by modulating antioxidant defenses, the methylglyoxal detoxification system, and proline metabolism [190]. The application of H_2_S not only boosts the antioxidant defense system (improving photosynthesis under salt stress, enhancing the activity of ABA, reducing glutathione) but also alleviates salt stress by coordinating stress response signal transduction pathways at the transcriptional level [191]. Hematin (a CO donor) application can reduce salt-induced oxidative damage in wheat seedling leaves by lowering H_2_O_2_ levels and lipid peroxidation, increasing chlorophyll content and antioxidant enzyme activity, and confirming CO as an effective antioxidant under abiotic stress [192]. Low concentrations of H_2_O_2_ applications have been proven to be effective against various stress factors. Pretreatment with H_2_O_2_ can enhance SOD activity in wheat seedlings, improving their tolerance to salt stress, while secondary treatment can alleviate stress and promote plant growth [193].

### 3.3. Beneficial Soil Microorganisms

Soil microorganisms commonly inhabit the rhizosphere of plants, among which, plant growth-promoting rhizobacteria (PGPR) play a pivotal role in sustainable agriculture. They enhance seed germination rates, increase the uptake of mineral nutrients, boost crop yields, and improve plant salt tolerance [194] (Figure 2). In contrast to the adverse effects of pesticides, fertilizer hormones, and other external substances on soil, the environment, and economic development, PGPR offers a benign method for increasing crop yields through both indirect and direct mechanisms [195]. The mechanisms of PGPR include inducing plant resistance to pathogens and adverse environments, regulating plant hormone levels, and dissolving nutrients to make them easily absorbed by plants [196,197].

## 4. Discussion and Expectations for the Future

Wheat, serving as a staple food for the majority of the global population, is a crucial source of carbohydrates and calories, boasting a long history and significant economic value. Salt stress emerges as one of the principal challenges to worldwide food production, with its severity escalating due to various natural phenomena and human activities. Resulting from oxidative stress and ion imbalance, salt stress severely hampers wheat seed germination, photosynthesis, respiration, nutrient uptake, and other physiological processes, ultimately leading to diminished yields or even to plant death. Research into the physiological and molecular mechanisms of wheat’s response to salt stress and the identification of strategies to mitigate its adverse effects can offer valuable insights for breeders and agronomists. This research aims to enhance wheat’s salt tolerance in the field and discover more effective and sustainable approaches to combat salt stress in wheat cultivation.

Breeding new salt-tolerant varieties and genetic improvement could fundamentally enhance the salt tolerance of wheat, with notable achievements already made [47,198]. The utilization of, and advancements in, modern techniques have facilitated a deeper understanding of wheat’s complex salinity traits. Moreover, the domestication of wild relatives and the introduction of beneficial genes are progressively being pursued. However, several challenges remain in this endeavor. First, the genetic transformation of wheat presents difficulties, with only a limited number of wheat varieties amenable to modification and low transformation efficiency. This limitation hampers the smooth transfer of essential salt-tolerant genes to target varieties. In response to this situation, researchers have developed growth regulatory factors (GRFS) and their cofactor interaction factors (GIFs) to improve the plant’s ability to regenerate and transform. TaGRF4-GIF1 improved the regeneration efficiency and regeneration speed of wheat and increased the convertible wheat genotypes. The combination of GRF4-GIF1 and CRISPR-Cas9 genome editing produced 30 wheat plants with the edited Q gene (AP2L-A5) destroyed [199]. The mTaGRF4-TaGIF1 complex significantly increases the range of wheat varieties available for conversion [200]. More regulation types and modes of regulators are still to be developed. Second, wheat strains developed under controlled laboratory conditions may not always exhibit the expected performance in field environments [201]. Under full-salinity treatment, a higher total weight indicates not only a higher salt tolerance, but also a higher yield potential [202], in which the mechanisms involved clearly warrant further attention. Additionally, the growth and yield of salt-tolerant varieties with different genotypes are influenced by soil nutrient levels and environmental conditions. The mechanism of wheat salt-tolerant germplasm and gene adaptation to field environments remains to be explored. Third, the safety and acceptance of genetically modified (GM) foods continue to be subjects of debate. With the increasing prevalence of GM foods, the safety evaluations and perceptions of GM foods vary significantly across different countries [203]. There is no doubt that more GM foods are coming to market, including wheat [204,205]. Fourth, due to the complex genome and changeable character regulation, many vital loci of wheat have yet to be accurately located. Achieving a deeper analysis of the whole wheat genome will require further in-depth research.

Many important questions remain to be explored. Are salt receptors present in plant membranes, cytoplasm, and organelles in addition to known and presumed ion receptors? How do salt stress responses interact with other environmental stresses? How can plant growth and yield be coordinated in salt-affected fields? The extent to which salinity damages plants depends on many different factors, including plant growth stage, ion level, genotype, temperature, the plant organs exposed to the salinity, the composition of the salinized solution, and the duration of salinity exposure. Is there a simple and efficient way to ignore all the differences and achieve the most favorable results? In combining artificial intelligence (AI) with wheat salt tolerance, modeling and forecasting based on existing basic knowledge may accelerate the progress of wheat salt tolerance research in the future. The growth of wheat will be significantly inhibited after salt stress. In this process, how will the transport of various organic nutrients and metabolite synthesis change in wheat? It is also important to identify genes related to nutrient utilization under salt stress.

Given wheat’s critical contribution to global food security, conducting research on wheat salt tolerance, and developing salt-tolerant varieties are invaluable for optimizing the use of saline–alkali lands and ensuring consistent food production. With ongoing advancements in scientific and technological methodologies, the mechanisms underlying wheat’s salt tolerance are becoming increasingly elucidated. This progress is expected to yield a wider array of salt-tolerant wheat varieties and facilitate the development of wheat germplasm capable of producing high and stable yields under saline–alkali conditions.

## Figures and Tables

**Table 1:** Transcription factors regulating salt stress in wheat.

TFs	Genes	Regulation	References
WRKY	*TaWRKY24*, *TaWRKY2*, *TaWRKY13*, *TaWRKY19*, *TaWRKY44*, *TaWRKY46*	positive	[76]
	*TaWRKY10*		[77]
AP2/ERF	*TaERF-6-3A*, *TaDREB3-AI*	negative	[78]
	*TaERF3*	positive	[79]
MYB	*TaMBY344*, *TaODORANT1*, *TaMYB33*, *TaMYB73*, *TaMYB86B*, *TaMYBsdul*, *TaMYB19*	positive	[80]
	*TaMYB64*		[81]
	*TaMYB32*		[82]
	*TaMYB32*		[83]
NAC	*TaNAC29*, *TaNAC2*, *TaNAC2a*, *TaNAC67*	positive	[84]
bZIP	*TabZIP60*	positive	[85]
	*TabZIP15*, *TabZIP14-B*		[86]
	*TabZIP8*, *TabZIP9*, *TabZIP13*		[87]
	*TaFDL2-1A*		[88]
bHLH	*TabHLH1*	positive	[89]
	*TabHLH39*		[90]
ZnFP	*TaZnFP*	positive	[91,92]

**Table 2:** Plant hormone signaling related genes involved in salt stress in wheat.

Hormone	Genes	Regulation	References
ABA	*TaASR1-D*	positive	[16]
	*TaABL1*		[127]
	*TaPYL5*		[24]
	*TaNCED3*		[128]
JA	*LOX*, *AOC*, *ACX*	positive	[129]
	*TaAOC1*		[130]
Auxin	*TaSAUR75*	positive	[131]
	*TaSAUR78*		[132]
	*TaLAX3-1B*		[133]
BR	*TaD11-2A*	positive	[134]
	*TaBZR1*		[135]
	*TaMADS22/47/55-4B*, *TaGRAS19-4B*, *TaBRD1-2A.1*		[136]
Ethylene	*TaACO1*	negative	[137]
	*TaACS6*, *TaACS7*, *TaACS8*	positive	[138]

## Data Availability

Data sharing is not applicable.
